# TNF-*α*/anti-TNF-*α* drugs and its effect on pregnancy outcomes

**DOI:** 10.1017/erm.2022.18

**Published:** 2022-06-10

**Authors:** Fang-fang Dai, Min Hu, Yu-wei Zhang, Rong-hui Zhu, Li-ping Chen, Zhi-dian Li, Yan-jie Huang, Wei Hu, Yan-xiang Cheng

**Affiliations:** 1 Department of Obstetrics and Gynaecology, Renmin Hospital of Wuhan University, Wuhan, Hubei 430060, China; 2 Department of Obstetrics and Gynaecology Ultrasound, Renmin Hospital of Wuhan University, Wuhan 430060, China

**Keywords:** Adverse pregnancy outcomes, anti-TNF-*α* drugs, TNF-*α* receptor, TNF-*α*

## Abstract

Pregnancy is a complex biological process. The establishment and maintenance of foetal–maternal interface are pivotal events. Decidual immune cells and inflammatory cytokines play indispensable roles in the foetal–maternal interface. The disfunction of decidual immune cells leads to adverse pregnancy outcome. Tumour necrosis factor (TNF)-*α*, a common inflammatory cytokine, has critical roles in different stages of normal pregnancy process. However, the relationship between the disorder of TNF-*α* and adverse pregnancy outcomes, including preeclampsia (PE), intrauterine growth restriction (IUGR), spontaneous abortion (SA), preterm birth and so on, is still indefinite. In this review, we thoroughly reviewed the effect of TNF-*α* disorder on pathological conditions. Moreover, we summarized the reports about the adverse pregnancy outcomes (PE, IUGR, SA and preterm birth) of using anti-TNF-*α* drugs (infliximab, etanercept and adalimumab, certolizumab and golimumab) currently in the clinical studies. Overall, IUGR, SA and preterm birth are the most common adverse pregnancy outcomes of anti-TNF-*α* drugs. Our review may provide insight for the immunological treatment of pregnancy-related complication, and help practitioners make informed decisions based on the current evidences.

## 1. Introduction

The successful implantation of semi-allograft embryos into the endometrium of the parent body without being rejected by the mother depends on the maintenance of inflammatory and immune microenvironment of the foetal–maternal interface. The establishment and maintenance of foetal–maternal microenvironment require the function of immune cells and diverse cytokines (Refs 1, 2). The function of decidual immune cells has been thoroughly investigated. However, the role of inflammatory cytokines, especially tumour necrosis factor (TNF)-*α*, in foetal–maternal interface is still indefinite. TNF-*α* is a pivotal cytokine in immunological and inflammatory conditions at the foetal–maternal interface. In the first trimester of pregnancy and childbirth period, TNF-*α* mediated moderate proinflammatory and immune response, contributing to the embryo transfer, trophoblast cell function and labour, while in the middle and late trimester of pregnancy inhibitory inflammatory response makes for embryonic development (Refs 3, 4). Emerging studies evidenced that the imbalance of TNF-*α* in the foetal–maternal interface was related to adverse pregnancy outcomes, including preeclampsia (PE), intrauterine growth restriction (IUGR), spontaneous abortion (SA), preterm birth, etc. (Refs 5, 6).

## 2. TNF-*α* and TNF-R-dependent signalling

TNF-*α* gene, locating on chromosome 6p21.3, spans approximately 3 kb and has four exons. The fourth exon encodes for more than 80% of the secreted protein. TNF-*α* mainly comes from activated macrophages and T-lymphocytes (Ref. 7). Two forms of TNF-*α* co-exist in mammals, which are soluble TNF-*α* (sTNF-*α*) and transmembrane TNF-*α* (mTNF-*α*) (Ref. 8). mTNF-*α*, namely TNF-*α* precursor (233 amino acid residues (26 kDa)), is mainly synthesised by activated macrophages and transferred onto the cytomembrane. Subsequently, mTNF-*α* is cleaved off by metalloproteinases or TNF-*α*-converting enzyme (TACE, also named ADAM17) to release sTNF-*α* (157 amino acid residues (17 kDa)) from cells in the form of monomer and trimer (Ref. 9). sTNF-*α* primarily binds with TNF-*α* receptors 1 (TNF-R1) to medicate inflammatory immune response, while mTNF-*α* mainly interacts with TNF-*α* receptors 2 (TNF-R2) to involve in the cellular proliferation, survival and other biological effects. Meanwhile, mTNF-*α* cannot only act as a ligand binding with TNF-Rs in the manner of cell–cell contact, but also serves as a receptor transmitting outside-to-inside (reverse) signals back into the mTNF-*α*-bearing cells (Ref. 10). TNF-R1 (55 kDa), containing conserved death-domain (DD) motifs, and constitutively expressed in various cells, can be bound and activated by sTNF-*α* and mTNF-*α*. Concomitantly, TNF-R2 (75 kDa) contains one or more TNF-R-associated factor (TRAF)-interacting motifs, mainly exists in restricted neurons, immune cells and endothelial cells, and can only be activated primarily by mTNF-*α* (Ref. 11). At the foetal–maternal interface, TNF regulates the function of trophoblast cells and immune cells, including cell apoptosis, proliferation, inflammation, immune and tissue remodelling in TNF-R1 and TNF-R2-dependent signalling manners (Refs 12, 13).

**Fig. 1.**
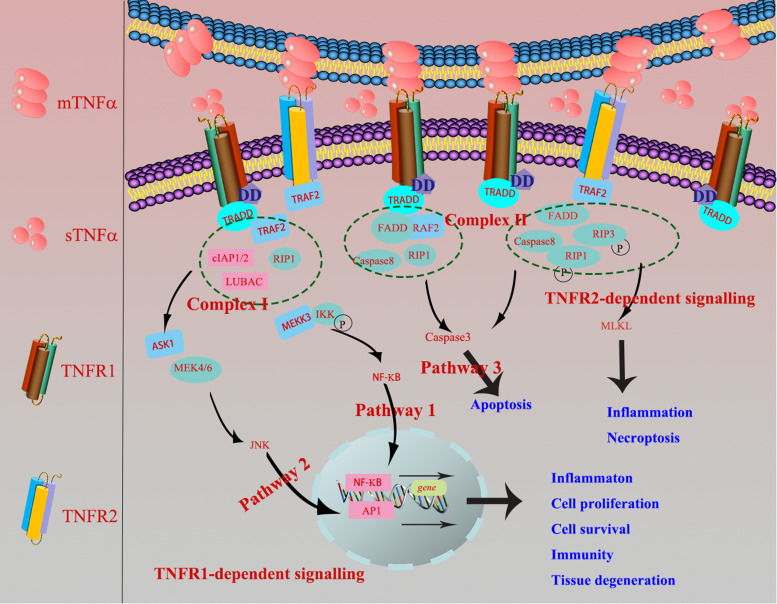
TNF-R1-dependent signalling: once TNF-R1 binds mTNF-*α* or sTNF-*α*, it recruits the adaptor protein TRADD or FFADD via its DD motifs. Subsequently, it recruits complex I, namely serine/threonine kinase receptor interacting protein-1 (RIP-1), TNF-R-associated factor 2 (TRAF-2), as well as cIAP1 and cIAP2. Then, it activates NF-*κ*B and JNK/AP1 signalling pathways and medicates the expression of targeted genes, finally involves in various biological processes, inducing inflammation, tissue degeneration, host defence, cell proliferation, cell survival and immunity. Besides, TRADD can also recruit and form the complex II (FADD, RIP-A, TRAF-2 and caspase 8) to finally activate caspase 3 eliciting cell apoptosis (Ref. 2). TNF-R2-dependent signalling: TNF-R2 is restricted to bind with mTNF-*α*, primarily recruiting TRAF-2 via its TRAF domain, which further causes the recruitment of complex II and activation of apoptosis, inflammation and necroptosis.

In terms of TNF-R1-dependent signalling, once binding with mTNF-*α* or sTNF-*α*, TNF-R1 recruits the adaptor protein TNF-R-associated DD (TRADD) or Fas-associating protein with a novel death domain (FFADD) via its DD motifs. Subsequently, it recruits complex I, namely serine/threonine kinase receptor interacting protein-1 (RIP-1), TRAF-2, as well as Cellular inhibitor of apoptosis 1 (cIAP1) and cIAP2. Then, it activates Nuclear factor kappaB (NF-*κ*B) and c-Jun N-terminal kinase/activator protien-1 (JNK/AP1) signalling pathways and medicates the expression of targeted genes, finally involves in various biological processes, inducing inflammation, tissue degeneration, host defence, cell proliferation, cell survival and immunity (Refs 14, 15). Besides, TRADD can also recruit and form the complex II (FADD, RIP-A, TRAF-2 and caspase 8) to finally activate caspase 3 eliciting cell apoptosis. When it comes to TNF-R2-dependent signalling pathway, TNF-R2, primarily recruiting TRAF-2 via its TRAF domain, is restricted to bind with mTNF-*α* (Fig. 1), and further recruited the complex II and activated apoptosis, inflammation, cell migration and necroptosis (Ref. 16).

## 3. TNF-*α* in adverse pregnancy outcomes

### 3.1 TNF-*α* and preeclampsia

PE is a characteristic complication of pregnancy and mainly occurs after 20 weeks of gestation, with a worldwide incidence rate of 5–8%. PE is one of the leading causes of maternal/foetal death during the gestation period (Refs 17, 18). The clinical manifestations of mothers are newly occurring hypertension and proteinuria, severely affecting the heart, liver, brain, lung, blood vessels and other organs (Refs 19, 20). Placental ischaemia and hypoxia induce the release of bioactive factors, including proinflammatory factors (TNF-*α*, interleukin (IL)-1*β*, IL-6, IL-12, interferon-*λ*), mediating extensive damages to the maternal vascular endothelium and causing the typical symptoms of PE. The pathogenesis of PE can be summarized as insufficient trophoblast invasion, disorder of systemic inflammatory response and immune regulation, and the dysfunction of angiogenesis and endothelial cells (Refs 21, 22).

Gelber *et al*. demonstrated that the increase of TNF-*α* may induce a secondary decrease in vascular endothelial growth factor (VEGF) and other angiogenic factors and eventually lead to placental dysplasia (Ref. 23). In addition, TNF-*α* can increase the cytotoxicity of natural killer cells (NK cells), trophoblast death and endothelial activation in the placenta, aggravate the dysfunction of endothelial cells, increasing adhesion molecules release and endothelial cells permeability in the cardiovascular system (Ref. 24). Another important role of TNF-*α* in PE patients is to stimulate B cells to produce autoantibodies against the angiotensin II type 1 (AT1) receptor. By binding and activating the AT1 receptor, TNF-*α* leads to the chronotropic response of heart, causing increased blood pressure (Ref. 25). In summary, the imbalance of foetal–maternal interface microenvironment caused by the change of TNF-*α* levels is closely related to the occurrence and development of PE.

### 3.2 TNF-*α* and intrauterine growth restriction

IUGR is defined as an impairment of the foetus's expected growth potential and an estimated foetal weight of less than 10% small for gestational age. Abnormalities in maternal nutrition, placental transport and the genetic potential of the foetus may lead to abnormal growth and development of the foetus.

Azizieh and Raghupathy *et al.* found that the expression of TNF-*α* in peripheral blood monocytes of IUGR patients was higher than that of normal pregnant women, illustrating the potential role of TNF-*α* in the occurrence of IUGR (Ref. 26). However, the specific mechanism of immunological studies about TNF-*α* in IUGR is limited. Foetal vascular disease is the pathogenic mechanism of IUGR. TNF-*α* directly damages endothelial cells, impairs the invasion and fusion of trophoblast cells (Refs 27, 28), and damages the remodelling of spiral arteries. In addition, TNF-*α* can also interfere with blood coagulation system, leading to placental thrombosis and aggravating placental hypoperfusion (Refs 29, 30). Therefore, these indicated that TNF-*α* is closely associated with IUGR and skeletal retardation. Elfayomy *et al*. demonstrated that Anti-Müllerian hormoneo (AMH), IL-6 and TNF-*α* in serum of IUGR were higher than that of normal pregnancy, which may serve as useful biochemical markers for IUGR. Nevertheless, reports about IUGR investigation are rare (Ref. 31). It is worth noting that more clinical studies are needed to identify them as diagnosis, treatment and prevention of biomarkers for IUGR. In mice, blocking TNF-*α* can improve spiral artery remodelling and pregnancy outcomes (Ref. 23). Therefore, TNF-*α* provides new options for the treatment and prevention of IUGR. At present, there have been only a few studies about the TNF family in IUGR, and the role of TNF in the pathogenesis of IUGR needs further investigation in the future.

### 3.3 TNF-*α* and spontaneous abortion

SA is one of the most common pregnancy complications, defined as the loss of a natural pregnancy before 20 weeks (Ref. 32). The epilogue of SA is complex and mainly includes genetic, anatomical, endocrine, immune, infection-related inflammation and psychological factors (Ref. 33). Given the postponement of the age of childbirth, changes in lifestyle and the influence of social and psychological factors, the incidence of SA is increasing year by year. The specific mechanism has not yet been fully clarified yet. In recent years, the relationship between the TNF-*α* and SA has attracted wide attention.

The abnormal expression of TNF-*α* is closely related to the occurrence of SA. For example, Jang *et al*. reported that TNF-*α* polymorphism is a genetic risk factor for SA, and TNF-*α*-863C > A variant is associated with an increased risk of SA (Ref. 34). In addition, the rate of SA in pregnant mouse models was observed to increase when the expression of TNF-*α* was upregulated (Ref. 35). At present, an increasing number of studies have shown that SA is associated with abnormal maternal immune function caused by the imbalance in Th1/Th2 and Th17/Treg ratio (Refs 36, 37). In addition, normal pregnancy involves complex interactions between the trophoblast, decidua and maternal immune cells. TNF-*α* also affects decidualization and function of decidual cells, thereby changing the microenvironment of foetal–maternal interface, thus mediating the occurrence of abortion. Fonseca *et al*. found that the high expression of TNF-*α* in decidual tissue of abortion can inhibit the differentiation of embryonic stem cells and impair decidualization, thus interfering with blastocyst implantation and/or pregnancy maintenance (Ref. 38). The latest research of Yang *et al*. showed that dNK cells highly express TNF-*α* in spontaneously aborted tissues, and TNF-*α* induces the upregulation of arylhydrocarbon receptor expression in dNK cells, thereby enhancing the cytotoxicity of decidual Natural Killer Cells (dNK cells) and making dNK cells develop an immune response to foetus, resulting in SA (Ref. 39). Further studies have found that reducing the cytotoxicity of dNK cells, thereby inhibiting the expression of Th1 cytokines, such as TNF-*α*, is conducive to creating a microenvironment at the foetal–maternal interface and maintaining pregnancy (Ref. 40). In addition, compared with normal pregnancy, the expression levels of TNF-*α* in peripheral blood and decidual tissue of patients with SA were abnormally increased, and TNF-*α* affects the function of decidual stromal cells by downregulating the expression of nucleotide oligomerisation domain 1, leading to SA (Refs 41, 42).

In conclusion, the abnormal upregulation of TNF-*α* expression can affect multiple key links in the normal pregnancy process. TNF-*α* mediates the occurrence of SA by affecting the balance of Th1/Th2 and Th17/Treg cells, endometrial decidualization, cytotoxicity of dNK cells and the function of decidual stromal cells. Further in-depth research on the specific mechanism of TNF-*α*-mediated SA will help reduce the incidence of SA and benefit women of childbearing age.

### 3.4 TNF-*α* and preterm birth

Preterm birth is defined as less than 37 full weeks or 259 full days of termination of pregnancy according to the WHO definition (Ref. 43). Generally speaking, preterm birth has a negative impact on the health of premature infants and causes a burden on the family of premature infants and society. Preterm birth is related to genetic factors, inflammation and immunity (Ref. 44). On the one hand, premature rupture of membranes (PROM) is one of the causes of premature delivery.

In a mouse model, upregulated TNF-*α* and IL-6 in placental tissue lead to PROM (Ref. 45). Wang *et al*. proved that the increase in the apoptosis of foetal membrane cells caused by TNF-*α* was related to PROM, further resulting in preterm birth (Ref. 46). On the other hand, abnormal uterine contractility also leads to premature birth. Many studies have demonstrated that TNF-*α* has a certain correlation with the contractility of the myometrium, mainly by affecting the expression of progesterone (PG) and Matrix Metallopeptidase 9 (MMP9). The increase in the expression and sensitivity of PG and MMP9 in the myometrium will lead to uterine contraction and delivery (Refs 47, 48). Specifically, TNF-*α* has been reported to reduce the activity and expression of NAD+-dependent 15-hydroxyprostaglandin dehydrogenase (PGDH) in trophoblast cells, resulting in maternal PE and premature delivery (Ref. 49). Recent advances have shown that the TNF family may regulate macrophage dysfunction leading to preterm birth, but the specific molecular mechanism needs further study.

### 3.5 TNF-*α* and other adverse pregnancy outcomes

Lan *et al*. reported that elevated levels of TNF-*α* and Th1/Th2 ratio are positive with embryo arrest rate (Ref. 50). Li *et al*. firstly found that TNF-*α* gene polymorphisms are associated with an increased risk of recurrent abortion (Ref. 51). Subsequently, other team further demonstrated the high expression of TNF-*α* in decidua tissue and peripheral blood of recurrent abortion (Ref. 41).

In conclusion, successful pregnancy requires foetal–maternal immune cross-talk between decidual immune cells and inflammatory cytokines. The dysregulation of TNF-*α* may leads to adverse pregnancy outcomes. During the pregnancy process, high TNF-*α* concentration not only increases the cytotoxicity of NK cells and stimulates B cells, but also breaks the balance of Th1/Th2 and Th17/Treg cells, increases trophoblast death, impairs its invasion and fusion, damages endothelial cells, and affects decidualization. Thus, above mechanisms induce pregnancy complications, namely, PE, IUGR, SA and preterm birth. Even though, the special mechanism of TNF-*α* needs further investigation.

## 4. Effect of anti-TNF-*α* drugs on adverse pregnancy outcomes

Rheumatoid arthritis (RA), inflammatory bowel disease (IBD), psoriatic arthritis (PsA), etc., are common inflammatory diseases. Women with chronic inflammatory diseases more likely suffer from adverse pregnancy outcomes. Specialists often propose that treatments are applied to limit the exacerbation of disease during pregnancy. For example, methotrexate (MTX) is the preferred drug for RA. However, MTX is a contraindicated drug during pregnancy owing to the teratogenicity and easily entering breast milk (Ref. 52). At present, anti-TNF-*α* drugs (infliximab (IFX), adalimumab (ADA), etanercept (ETA), golimumab and certolizumab) have been widely applied to clinical inflammatory diseases, such as Crohn's disease, ulcerative colitis, RA and so on. However, the influence of those drugs on adverse pregnancy outcomes for pregnant women with inflammatory diseases has hardly been explored. On the one side, the anti-TNF-*α* drugs cannot be interrupted especially in the acute phase of the inflammatory diseases. On the other side, inflammatory diseases are easily lead to adverse pregnancy outcomes. Therefore, it is indispensable and worthy to statistic and analyze the effect of anti-TNF-*α* drug on the adverse pregnancy outcomes. In this review, we summarize the reports about the adverse pregnancy outcomes (PE, IUGR, SA and preterm birth) of using anti-TNF-α drugs (infliximab, etanercept and adalimumab, certolizumab and golimumab) currently in the clinical studies (Table 1).

**Table 1.** The statistics of anti-TNF*α* drug (infliximab, adalimumab, etanercept, golimumab and certolizumab) on the pregnancy outcome (live birth, PE, IUGR, SA, preterm birth and other pregnancy outcomes)

Anti-TNF-*α*	Pregnancy outcome						Abortion rate		Number	Disease	References
	Live birth	PE	IUGR	SA	Preterm birth	Other	1st trim	2nd/3rd trim			
Infliximab	1526/1850	No report		12.1%	9.2%	Congenital anomalies (2.0%)	No report	No report	1850	CD/UC	(Ref. 54)
Infliximab	64/96	No report		14/96	No report		No report	No report	96	RA/CD	(Ref. 55)
Infliximab	10/10	No report	No report	No report	3/10		No report	No report	10	IBD	(Ref. 56)
Infliximab	32/42	No report	No report	No report	No report		No report	No report	42	IBD	(Ref. 65)
Infliximab	1740/2017	No report	No report	No report	104/1754		No report	No report	2017	IBD	(Ref. 81)
Infliximab	220/252: 69/72	No report	No report	28/252; 3/72	No report		No report	No report	252 (maternal); 72 (paternal)	CD	(Ref. 57)
Infliximab	413/495	No report	No report	43/495	63/407		No report	No report	495	IBD	(Ref. 82)
Infliximab	46/59	No report	No report	10/59	8/59		No report	No report	59	IBD	(Ref. 83)
Infliximab	155/194	No report	No report	15/194	21/194		No report	No report	194	IBD	(Ref. 72)
Infliximab	117/151	No report	No report	11/151	16/151		No report	No report	151	IBD	(Ref. 72)
Infliximab	55/82	No report	No report	11/82	No report		No report	No report	82	CD	(Ref. 55)
Infliximab	No report	No report	No report	No report	7/83		No report	No report	83	IBD	(Ref. 84)
Infliximab	4/17	No report	No report	1/17	No report		No report	No report	17	RA	(Ref. 85)
Infliximab	67/83	No report	No report	9/83	16/66		No report	No report	83	IBD	(Ref. 86)
Adalimumab	65/74	No report	No report	No report	No report		40%	2nd:16%, 3rd:44%	74	RA	(Ref. 61)
Adalimumab	No significant differences were found in pregnancy and foetal outcomes when compared to the general population						7/7	2nd:4/7	7	IBD	(Ref. 65)
Adalimumab	24/28	No report	No report	2/28	4/24		No report	No report	28	IBD	(Ref. 87)
Adalimumab	45/53			5/53	2/53	Stillbirth (1/53)				CD/UC/RA	(Ref. 88)
Adalimumab	35/47	No report	No report	No report	No report	No report	No report	No report	41＋6 (immunoglobulin and non-immunoglobulin)	RMA and implantation failure	(Ref. 89)
Adalimumab	221/257	No report	No report	25/257	24/213	Teratogenesis: 22/221	No report	No report	257	RA/ CD	(Ref. 62)
Etanercept	2169/3048	No report	No report	590/3048	No report	Artificial abortion: 265/3048	No report	No report	3048	CIA	(Ref. 90)
Etanercept	66%	No report	No report	20%	No report	Teratogenesis (Ref. 1)	No report	No report	56	CIA	(Ref. 58)
Etanercept	1354/1835	No report	No report	306/1835	No report	Artificial abortion: 168/1835	No report	No report	1835	PsA	(Ref. 90)
Etanercept	16/17	No report	No report	2/17	No report		No report	No report	17	RA/ankylosing spondylitis/PsA	(Ref. 91)
Etanercept	No report	No report	No report	No report	19/106		No report	No report	140	RA	(Ref. 82)
Etanercept	No report	No report	No report	2/15	1/15		No report	No report	15	RA	(Ref. 70)
Etanercept	No report	No report	No report	0/10	1/10		No report	No report	10	RA	(Ref. 70)
Etanercept	85/95	No report	No report	10/95	No report	Gestational hypertension: 1/95	No report	No report	95	Refractory innate immunity RMA	(Ref. 74)
Etanercept	6/6	No report	No report	0/6	No report		No report	No report	6	SpA	(Ref. 73)
Etanercept	1/1	No report	No report	0/1	No report		No report	No report	1	JIA	(Ref. 73)
Etanercept	0/1	No report	No report	1/1	No report		1/1	No report	1	PsA	(Ref. 73)
Etanercept	0/2	No report	No report	1/2	No report	Early termination of pregnancy (1/2)	2/2	No report	2	RA	(Ref. 73)
Etanercept	2/2	No report	No report	No report	No report		No report	No report	2	RA	(Ref. 85)
Golimumab	143/208	No report	4/208	47/208	5/208		38/183	0/183	208	RA and IBD	(Ref. 92)
Golimumab	No report	No report	No report	32.5%	No report		No report	No report	40	UC	(Ref. 93)
Certolizumab	26/47(55.3%)	No report	No report	No report	No report		No report	No report	No report	RA	(Ref. 93)
Certolizumab	459/538	5/538	5/538	47/538	No report	Serious maternal infections (Ref. 22), gestational diabetes (Ref. 6), induced abortion (Ref. 27), stillbirth (Ref. 5)	No report	No report	538	RA/ CD	(Ref. 69)
Certolizumab	No report	No report	No report	2/25	2/25	Premature rupture of the membranes (Ref. 9), light-for-date (Ref. 7)	No report	No report	25	RA	(Ref. 70)
Certolizumab	254/339	6/339	6/339	84/339	No report	Stillbirth (Ref. 1), placental abnormalities (Ref. 4), infection (Ref. 5), disease flare (Ref. 10), gestational diabetes (Ref. 5), congenital malformation (Ref. 8)	No report	No report	339	RA (118), CD (192), other (Ref. 29)	(Ref. 71)
Certolizumab	8/17	No report	No report	1/17	No report	Low birth weight (1/17)	No report	No report	17	IBD	(Ref. 72)

PE, preeclampsia; IUGR, intrauterine growth restriction; SA, spontaneous abortion; CD, Crohn's disease; UC, ulcerative colitis; RA, rheumatoid arthritis; IBD, inflammatory bowel disease; IFX, infliximab; ETA, etanercept; ADA, adalimumab; CZP, certolizumab-pegol.

### 4.1 Infliximab and adverse pregnancy outcomes

IFX is the first anti-TNF-*α* drug to be approved to apply to IBD, PsA and RA. Truta and Geldhof *et al.* reported that preterm births and IUGR rate are increased in the IFX group (*P* < 0.049) (Refs 53, 54). Katz *et al.* collected the outcome data of 96 women who were exposed to IFX, the result showed that live birth rate is 67% (64/96), the percent of SA is 15% (14/96) and termination of treatment is 19% (18/96) (Ref. 55). Geldhof *et al*. retrospectively analyzed the Janssen's global safety surveillance databases and the pregnancy outcomes of 1725 patients exposed to IFX. In total, 1549/1875 (82.61%) were reported as live births, 226/1875 (12.1%) were reported as RA, 143/1549 (9.2%) were reported as preterm births, and the prevalence of IUGR is ⩽1.1% (Ref. 54). However, a retrospective study by Mahadevan *et al*. reported that three infants were preterm birth and one is low-birth weight in all 10 pregnancies that exposed to IFX (Ref. 56). In those data, there is no doubt that some pregnant women choose early termination of pregnancy. Lichtenstein *et al.* analyzed the data in the TREAT Registry (1999–2012) and concluded that the rates of miscarriage (10% *versus* 6.7%) and neonatal complications (6.9% *versus* 10%) have no statistic difference in IFX exposure and IFX non-exposure patients (Ref. 57). Overall, IFX may increase the risk of preterm birth, caesarean delivery, and low birth weight, teratogenesis and stillbirth are rarely reported (Refs 53, 58).

IFX, an immunoglobulin antibody, has difficulty in passing through the maternal placenta during the first trimester of pregnancy, while at the end of the second and the third trimesters, it often transfers through the placenta. IFX has been detected in serum of child at 6 weeks of age, whose mother was exposed to IFX (Ref. 59). Another case reported that all eight patients with IFX treatment delivered healthy children, and the IFX levels were higher in infant than that of in mother. Besides, the IFX levels take 2–8 months to be completely cleared owing to the immature reticuloendothelial system. In fact, Julsgaard *et al.* detected IFX exists in infants until 12 months of age (Ref. 60). Hence, the use of IFX during pregnancy is a worthy consideration. Of course, these studies arised from differences in their design and small sample size often incites controversy surrounding. But current guidelines propose that pregnant women are advised to terminate IFX exposure in the third trimester of pregnancy (Ref. 61).

### 4.2 Adalimumab and adverse pregnancy outcomes

Prospective controlled observational cohort study from the University of California San Diego showed that the live birth of ADA-exposed group is 221/257 (85.9%), which is comparative to that in the healthy pregnancy group [198/225 (88%)]. In the live birth, infants of 22/221 (10%) ADA-exposed women in the first-trimester were diagnosed with a major birth structural defect (OR 1.10, 95% CI 0.45–2.73) while this ratio in healthy group was 12/198 (6.1%) (OR 1.43, 95% CI 0.33–6.27). Meanwhile, HR for SA in ADA-exposed group was higher than that of healthy group (95% CI 0.96–18.95). The reason may be that ADA-exposed group are more likely to terminate pregnancy considering the potential harm for fetus. Besides, the likelihood of preterm birth of patients in ADA-exposed group is higher than that of in healthy group (HR 2.59, 95% CI 1.22–5.50) (Ref. 62). A prospective study compared the level of TNF-*α* in pregnant women between ADA-treatment group and IFX-exposed group. It manifested that at birth, ADA in cord blood of pregnant women with ADA-treatment group was obviously lower than IFX-exposed group, and in third stage pregnancy, the transportation of IFX is exponential in the placenta while the linear growth transfer of ADA is restricted, which implied that the residual ADA is more likely to reduce the relapse risk of mother (Ref. 63). Interestingly, Winger *et al.* reported that ADA treatment can improve pregnancy rates of women undergoing IVF via change the ratio of Th1 and Th2 (Ref. 64). ADA treatment has been reported that there are no significant differences in pregnancy and foetal outcomes when compared to the general population (Ref. 65). They can induce SA and preterm birth in three different trimesters of gestation as the medicine of RA (Refs 61, 62, 66).

### 4.3 Certolizumab and adverse pregnancy outcomes

Certolizumab-pegol (CZP) is mainly used for patients with severe plaque psoriasis. CZP is a pegylated humanised anti-TNF-*α* antigen-binding fragment, and cannot bind with the Fc receptor of neonatal (Refs 64, 67). Thus, penetration of placenta, breast milk content, and oral bioavailability are all low (Ref. 68). At present, there are few large-scale data about the pregnancy outcomes of CZP exposure. Clowse *et al*. reported that of 538 patients with known outcomes of CZP exposure, 459 were live births, 47 were SA and 8 were congenital malformations according to the prospective and retrospective data from the UCB Pharma safety databases (Ref. 69). Besides, the serious maternal infections, gestational diabetes, stillbirth, premature rupture of the membranes, stillbirth, placental abnormalities, infection, disease flare, gestational diabetes, congenital malformation and low birth weight have also been reported in the process of CZP for the therapy of RA and IBD (Refs 69–72).

### 4.4 Etanercept/golimumab and adverse pregnancy outcomes

Apart from IBD, PsA and RA, ETA has been widely used in chronic inflammatory arthritis, juvenile idiopathic arthritis and ankylosing spondylitis. SA and preterm birth are the most common adverse pregnancy outcomes, and SA often occurs in the first trimester of pregnancy (Ref. 73). Noteworthly, one gestational hypertension has been reported in the treatment of refractory recurrent SA (Ref. 74). The data from Italian rheumatology centres show that the live births were 66%, and the SA was 20% in the 79 exposed pregnancies, including 56 pregnant women exposed to ETA, 13 to ADA, 3 to IFX, 2 to CZP, etc. (Ref. 58). Golimumab was approved by the FDA in April 2009 as a treatment for moderately to severely active RA in combination with MTX (Ref. 58). The report of golimumab is relatively less. It is no doubt that both golimumab and certolizumab can induce IUGR, SA and preterm birth.

Overall, the most common adverse pregnancy of above anti-TNF-*α* drugs is IUGR, SA and preterm birth. ETA and golimumab mainly induce early abortion, while ADA increases the risk of abortion in any phase of pregnancy. Apart from certolizumab, no case about PE complication of other drugs is reported. Teratogenesis and stillbirth are mainly related to IFX and certolizumab. One report about gestational hypertension has been proposed in the use of ETA. The live birth of both drugs is relatively high. How to balance the advantages and disadvantages of the drugs and which medications should be chosen at different trimester of pregnancy have no consensus in the clinical studies. Our review may aid clinicians and women of childbearing age in their inflammatory disease's treatment.

### 4.5 Effect of anti-TNF-*α* drugs on immune system suppression

Anti-TNF-*α* drugs can decrease the activation of NK cells. Nocturne *et al*. reported that NK cell function was impaired in the anti-TNF group compared with MTX group during the treatment of RA (20.9 *versus* 31.3%, *P* = 0.04) (Ref. 75). Defendenti *et al*. detected B-cell subsets in bone marrow and peripheral blood of IBD patients, which showed that anti-TNF-*α* drugs inhibit the production of B cells in bone marrow and have no influence in circulating B cells (Ref. 76). Anti-TNF-*α* drugs treatment significantly decreased circulating Th17, Tfh and other pro-inflammatory immune cells, but highly increased circulating Treg and Breg levels (Refs 77, 78). In addition, anti-TNF-*α* drugs can inhibit proinflammatory macrophages polarisation (Ref. 79). The above immune system suppression from anti-TNF-*α* drugs may well explain the adverse pregnancy outcomes.

## 5. Future challenges

It has been demonstrated that high disease activity has been found to have little effect on pregnancy outcomes of IUGR, while the potential use of anti-TNF-*α* drugs can control disease outbreaks and severity during pregnancy (Ref. 80). At present, there is a lack of consensus on recommendations for the use of these drugs during pregnancy. Hence, it is a big challenge to carefully weigh the benefits and risk of anti-TNF-α drugs exposed to the mother and infant. The problem and puzzle about anti-TNF-*α* drug on the pregnancy outcome are whether the drug induces infertility (Ref. 1); whether the drug causes adverse pregnancy outcomes in different time of conception (Ref. 2); whether the drug can lead to foetal abnormalities and other potential complications (Ref. 3). Large-scale and long-time clinical data on its impact on pregnancy outcomes are indispensable in the future.
